# Nanomaterials-Functionalized Hydrogels for the Treatment of Cutaneous Wounds

**DOI:** 10.3390/ijms24010336

**Published:** 2022-12-25

**Authors:** Yangkun Liu, Gongmeiyue Su, Ruoyao Zhang, Rongji Dai, Zhao Li

**Affiliations:** 1 Institute of Engineering Medicine, School of Medical Technology, Beijing Institute of Technology, 5 South Zhongguancun Street, Haidian District, Beijing 100081, China; 2 Beijing Key Laboratory for Separation and Analysis in Biomedicine and Pharmaceuticals, Beijing Institute of Technology, 5 South Zhongguancun Street, Haidian District, Beijing 100081, China; 3 School of Life Science, Beijing Institute of Technology, 5 South Zhongguancun Street, Haidian District, Beijing 100081, China

**Keywords:** nanomaterials, functionalization, hydrogels, treatment, cutaneous wounds

## Abstract

Hydrogels have been utilized extensively in the field of cutaneous wound treatment. The introduction of nanomaterials (NMs), which are a big category of materials with diverse functionalities, can endow the hydrogels with additional and multiple functions to meet the demand for a comprehensive performance in wound dressings. Therefore, NMs-functionalized hydrogels (NMFHs) as wound dressings have drawn intensive attention recently. Herein, an overview of reports about NMFHs for the treatment of cutaneous wounds in the past five years is provided. Firstly, fabrication strategies, which are mainly divided into physical embedding and chemical synthesis of the NMFHs, are summarized and illustrated. Then, functions of the NMFHs brought by the NMs are reviewed, including hemostasis, antimicrobial activity, conductivity, regulation of reactive oxygen species (ROS) level, and stimulus responsiveness (pH responsiveness, photo-responsiveness, and magnetic responsiveness). Finally, current challenges and future perspectives in this field are discussed with the hope of inspiring additional ideas.

## 1. Introduction

The skin is the largest organ of the body and performs secretory, excretory, and absorptive functions. It can also be used to sense touch, temperature, pain, and itches [1]. The human body will be tremendously affected by skin damage [2,3,4]. The imperative need for rapid clinical progress in the treatment of skin wounds is urgent, and incorrect care can lead to irreversible consequences that can have a huge impact on patients [5,6]. Human skin is capable of some self-healing, but the long-term repair process inevitably accompanies chronic wound infections, which in turn impede the healing process [7]. Therefore, it is necessary to develop effective in vitro tissue engineering materials to promote rapid skin wound healing [8]. Ideal wound dressings need to be biocompatible, adhere to the wound tightly, stop the bleeding for several hours, provide a suitable environment for healing, be antimicrobial, create a barrier from the outside environment, and be easily applied [9,10,11]. Currently, numerous forms of dressings have been developed to accelerate skin wound healing, such as scaffolds [12,13,14], electrospinning nanofibers mats [15,16,17], sponges [18,19,20], and hydrogels [21,22,23]. Indeed, hydrogels have drawn great attention in recent years [24,25].

Hydrogels are crosslinked 3D networks that swell with large amounts of water [26,27]. They have been widely used in dressings because they can keep the wound moist, cool the wound, absorb inflammatory exudates from the wound, provide a breathable microenvironment, prevent anaerobic bacteria from colonizing, and release preloaded therapeutic agents [28,29,30,31]. However, the biological efficiency of biomaterials is typically insufficient for multifunctional applications when they are created as hydrogel matrix alone. Thus, a common way to improve the comprehensive performance of hydrogels as wound dressings is to introduce modifiers for targeted functionalization.

Nanomaterials (NMs) are tiny particles, ranging in size from 1 to 100 nm [30,32]. They have been widely used in various fields, such as catheter coatings, antimicrobial agents, and biomarker detection [33,34,35]. Additionally, they play irreplaceable roles in hydrogels used for cutaneous wound treatment, even outperforming the biological efficacy of drugs in some aspects. A nanometer-level scale can endow the materials with various superiorities: (1) NMs can generate new or enhanced electricity, light, heat, and magnetism; (2) they have stronger penetrating power to destroy structures of bacteria and viruses than conventional wound healing drugs or materials with larger scales; (3) they can change the pharmacokinetics and drug delivery efficiency; (4) the shape-tunable properties of NMs and the specific surface area can provide more binding sites for the attachment of bioactive substances such as drugs, nucleic acids, and proteins [36,37,38,39,40]. Recently, a variety of NM-loaded hydrogels with diverse functionalities have been developed as advanced wound dressings. The hydrogel can be endowed with the inherent properties of NMs. For NMs, hydrogels can offer a stable environment. Moreover, they can also avoid the explosive release and accumulation of NMs in wounds, which may result in biotoxicity [41]. The combination of NMs and hydrogels provides more opportunities for the treatment of skin wounds. Therefore, a systemic summary of this field will benefit its development.

Herein, the progress of NMs-functionalized hydrogels (NMFHs) in cutaneous wound treatment over the past five years is reviewed. Firstly, the fabrication strategies of the NMFHs are summarized and illustrated. Then, functions brought by NMs to the NMFHs are overviewed. Furthermore, the current research challenges and prospects of this field are also discussed.

## 2. Fabrication Strategies

**Figure 1:**
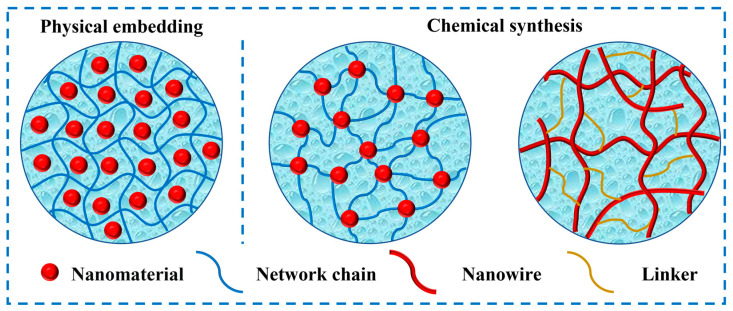
Schematic of fabrication strategies of physical embedding and chemical synthesis of NMFHs.

Based on the mode of introduction of NMs into hydrogel networks, fabrication strategies for NMFHs can be mainly divided into two categories, which are physical embedding and chemical synthesis (Figure 1). For the former, no covalent bonds exist between NMs and gel network chains. For the latter, NMs form covalent bonds with gel networks. These NMs usually play the role of crosslinking points. In addition, some NMs with a specific state, such as nanowires, can also play the role of chains to form the network through covalently linking by linkers.

### 2.1. Physical Embedding

**Figure 2:**
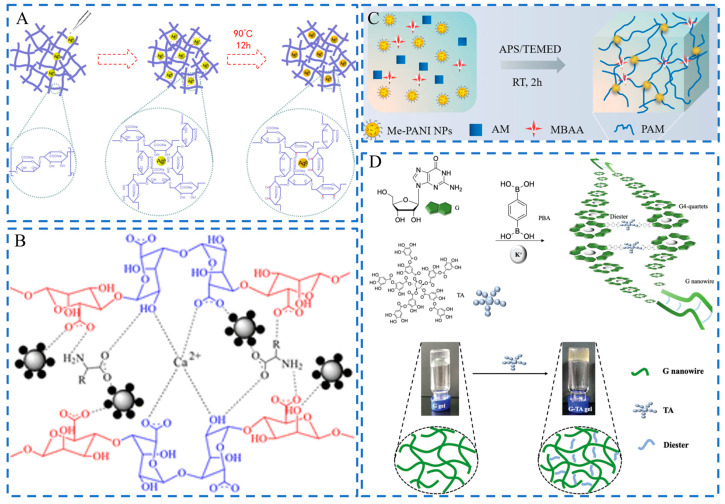
Schematics of fabrications of NMFHs by physical embedding and chemical synthesis. (**A**) Formation of an NMFH through embedding Ag NPs in the PVA/SA/CMCS-based network. Adapted with permission [42]. Copyright 2020, Elsevier. (**B**) The network structure of the NMFH is based on SA, Ag@TiO_2_ NPs, amino acids, and Ca^2+^. Adapted with permission [44]. Copyright 2020, The American Chemical Society. (**C**) Schematic of the formation of the NMFH from Me-PANI NPs, AM, and *N*,*N’*-methylenebisacrylamide (MBAA) through free radical copolymerization. Adapted with permission [45]. Copyright 2021, Wiley-VCH. (**D**) Schematic of the formation of the NMFH based on G NWs, K^+^, TA, and PBA. Adapted with permission [46]. Copyright 2022, The Royal Society of Chemistry.

Physical embedding is the most common and convenient strategy to fabricate NMFHs. For example, Xu and Chen et al. constructed an antimicrobial hydrogel dressing via loading Ag nanoparticles (NPs) into a poly(vinyl alcohol) (PVA)/sodium alginate (SA)/carboxymethyl chitosan (CMCS) (PVA/SA/CMCS)-based network [42]. As reducing agents, hydroxyl and carboxyl groups on sodium alginate (Alg) chelated Ag^+^ which was reduced to Ag^0^ to form the Ag NPs-embedded matrix (Figure 2A). NMs can also form non-covalent interactions with gel network chains, such as electrostatic interaction and hydrogen bonds. Xia et al. designed a nanocomposite hydrogel based on Alg and edaravone-loaded Eudragit NPs (EDA NPs). Eudragit NPs were positively charged polymeric NMs, which could bind to negatively charged Alg chains through electrostatic interactions to form the hydrogel [43]. Keleher et al. prepared an antimicrobial hydrogel dressing by introducing Ag-coated TiO_2_ NPs (Ag/TiO_2_ NPs) into an SA-based matrix [44]. Due to the existence of many hydroxyl groups on the NPs core (TiO_2_), hydrogen bonds formed between the NPs and alginate chains. This non-covalent interaction results in a higher packing density (Figure 2B).

### 2.2. Chemical Synthesis

The NMs bonded to gel networks by covalent bonds are usually more stable than the physically embedded ones. The strategy of chemical synthesis has been applied to the fabrication of NMFHs through the introduction of organic NPs or functionalized inorganic NPs into gel networks. For example, Pang and Zhu developed a photothermal antibacterial hydrogel through introducing double bond-ended polyaniline NPs (Me-PANI NPs) into the polyacrylamide (PAM) network [45]. The Me-PANI NPs were self-assembled from the amphiphilic copolymer PANI-*g*-Methacrylated glycol chitosan (MeCC) which was formed via grafting the hydrophobic PANI onto the hydrophilic MeGC chain. The NPs acted as the crosslinking points to form the hydrogel through free radical copolymerization among double bonds on the NPs and acrylamide (AM) (Figure 2C). NMs with specific states can also act as network chains to form hydrogels through covalent linking by linkers. This is a kind of special chemical synthesis approach. Zhao and Chen et al. fabricated a hydrogel based on guanosine nanowires (G NWs), tannic acid (TA), 1,4-phenylenediboronic acid (PBA), and K^+^ through a one-pot method [46]. G NWs were first created through the hierarchical assembly of guanosine and K^+^. Then the hydrogel was fabricated from G NWs, TA, and PBA through the formation of borate esters between hydroxyl groups on G NWs, TA, and boronic acid groups on PBA (Figure 2D).

## 3. Functions

**Figure 3:**
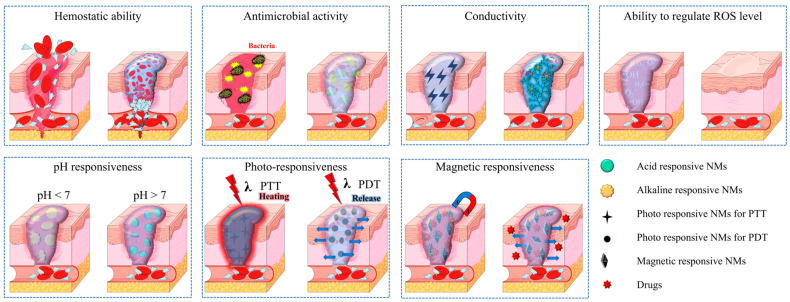
Schematic of functions of NMFHs for the cutaneous wound treatment. (PTT: photothermal therapy; PDT: photodynamic therapy).

In this section, NMFHs in cutaneous wound treatment are overviewed from the perspective of the function NMs bring to the hydrogels. Five categories of functions which are hemostasis, antimicrobial activity, conductivity, regulation of reactive oxygen species (ROS) level, and stimulus responsiveness (pH responsiveness, pho-to-responsiveness, and magnetic responsiveness) are involved (Figure 3).

### 3.1. Hemostatic Ability

The primary therapeutic treatment method for penetrating wounds is hemostasis, which acts in the initial phase of the wound. A critical aspect of healing cutaneous wounds is maintaining hemostasis. [47,48,49]. Patients suffering from penetrating wounds require rapid hemostatic treatment in order to avoid serious complications, such as amputation and death [50]. The process of hemostasis consists of three synergistically ordered processes: vasospasm and hemostasis, the formation of platelet embolism, and the coagulation cascade [51]. The ideal hemostatic materials should be able to adhere to tissues quickly, be removed at the wound site, and keep long-term stability. Additionally, hemostatic hydrogels should also have strong mechanical strength to resist blood pressure [51,52].

**Figure 4:**
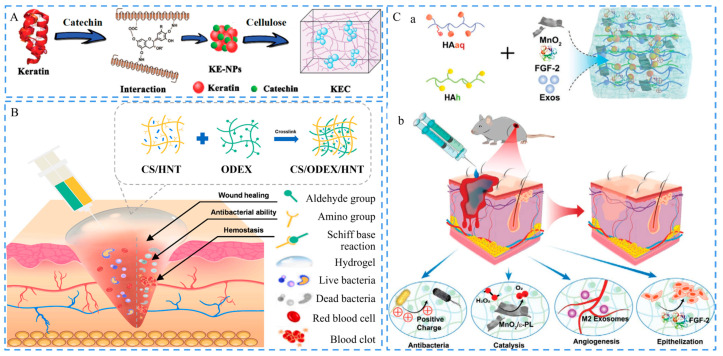
Schematics of hemostatic hydrogels. (**A**) Schematic of molecular assembly of keratin-catechin (KE)-NPs and formation of the cellulose/keratin-catechin composite hydrogel (KEC). Reproduced with permission [54]. Copyright 2018, Royal Society of Chemistry. (**B**) Schematic of the CS/ODEX/HNT hydrogel. The hydrogel was prepared in situ by the crosslinking between CS and ODEX via the Schiff base reaction and showed homeostatic and antibacterial abilities. Reproduced with permission [55]. Copyright 2021, Elsevier. (**C**) Preparation and application of the HA@MnO_2_/FGF-2/Exos hydrogel. (a) The preparation procedure for the hydrogel. (b) The hydrogel could promote wound healing by killing bacteria, catalyzing H_2_O_2_ decomposition, and accelerating neovascularization and re-epithelialization. Adapted with permission [63]. Copyright 2021, Wiley-VCH.

Li et al. developed a keratin-catechin nanocomposite modified cellulose hydrogel, as presented in Figure 4A. The catechin crosslinked the keratin and then clustered into nanoparticles (NPs), which were embedded in a cellulose-based hydrogel for the treatment of a full-thickness skin defect. The catechin endowed the hydrogel with strong adhesiveness [53], while keratin had hemostatic potential by accelerating thrombin activation and inducing platelet aggregation. They synergistically achieved rapid hemostasis [54]. Non-metallic minerals with excellent biological performances have also been used in the development of hemostatic hydrogels. Halloysite nanotubes (HNTs) are natural clays with good hemostatic and cell proliferation promotion abilities. Lu and Zhang et al. introduced them into a chitosan (CS)/oxidized dextran (ODEX)-based hydrogel to reduce hemostatic time and blood loss (Figure 4B). This hydrogel was formed by in situ crosslinking through the Schiff base reaction between amino groups (-NH_2_) in CS and aldehyde groups (-CHO) in ODEX. Besides, the composite pre gel solution could transfer to hydrogel within 1 s after injection, which could adapt to wounds with different shapes [55]. Cuttlefish melanin is a natural biomaterial with a catechol structure. It can significantly improve the adhesion performance of hydrogels, which is a crucial property that hemostatic materials ought to have [56,57]. Guo et al. developed a composite hydrogel based on adipic dihydrazide-modified hyaluronic acid (HA), benzaldehyde-functionalized poly(ethylene glycol)-*co*-poly(glycerol sebacate) (PEGSB), and cuttlefish melanin NPs [58]. This hydrogel exhibited great adhesive and hemostatic abilities, indicating potential for motion wound treatment in clinics.

In addition to non-metallic NPs, certain metallic NPs and their composites have been used in the development of hemostatic hydrogels. Lan et al. introduced Ag NPs into a CS/gelatin/sodium hyaluronate (SH)-based hydrogel to prepare a composite hydrogel that could control hemorrhage and promote wound healing after hemostasis surgery [59]. Ag NPs could achieve rapid hemostasis by promoting phosphatidylserine exposure, thrombin formation, and inducing platelet aggregation by activating positively charged surfaces [60,61]. Lee et al. mixed Ag@metal organic framework (Ag@MOF) with graphene oxide (GO) and loaded them into silk glue/CS-based hydrogels to affect red blood cells and platelets, accelerating thrombus formation, thus improving coagulation [62]. Xiao, Feng, Mi, and Liu et al. synthesized hydrazide-grafted HA (HAh) and aldehyde and quaternary ammonium-grafted HA (HAaq) to apply them in a permeable HA@MnO_2_/fibroblast growth factor 2 (FGF-2)/exosomes (Exos) hydrogel fabrication with rapid hemostatic function (Figure 4C). The positively charged quaternary ammonium group on the hydrogel could attract blood cells by the electrostatic interaction and improve the coagulation ability, while MnO_2_-*ε*-polylysine (*ε*-PLys) nanosheets (NSs) in the hydrogel network could catalyze the conversion of excessive hydrogen peroxide (H_2_O_2_) generated at the wound to O_2_, thus removing ROS and providing O_2_ for wound healing [63]. Jayakumar et al. developed a composite nano bio glass that was loaded into a CS hydrogel to further enhance its coagulation function. The silica, calcium, and phosphate in nano bioglass synergized with CS to achieve rapid bleeding control by activating coagulation factor XII and the intrinsic and exogenous pathways [64].

### 3.2. Antimicrobial Activity

Inhibition of bacterial infections is a primary factor in skin wound care. There is an urgent need to develop advanced materials with antimicrobial activity [65,66]. Some biopolymers used to prepare hydrogels to have inherent antimicrobial properties, such as CS [67], chitin [68], Alg [69], and bacterial cellulose [70]. The antimicrobial mechanism of these biomaterials could contribute to the destruction of the surface structure of the bacteria by the biological materials, resulting in the outflow of the fluid in the cells or the coating of the bacteria, resulting in cell death [71,72]. In addition, some PEG-based hydrogels [73], cationic peptide-based hydrogels [74], and zwitterionic hydrogels [75] have antifouling properties and can promote antimicrobial activity by inhibiting bacterial adhesion. However, the antibacterial activity of the above hydrogels is limited, and the antifouling hydrogels only act as a barrier against unfriendly factors. It is necessary to introduce ingredients with antibacterial activity. Many studies have introduced antibiotics to improve the antibacterial activity of hydrogels, such as tetracycline [76], gentamicin [77], amoxicillin [78], and ciprofloxacin [79]. Although antibiotics have been widely used in clinical treatment, the increasing severity of drug resistance should not be ignored. Therefore, it is meaningful to develop new antibacterial strategies [2]. NMs are considered to be effective alternatives to antibiotics and there are many reports about the introduction of NMs as antibacterial agents or antibacterial enhancers into hydrogels.

For instance, Ag NPs are the most widely used metal NMs in the antibacterial materials development because they can effectively resist almost all types of infections [80]. The antibacterial mechanism of Ag NPs is evident. (1) They can directly adhere to the cell wall and cell membrane, causing obvious morphological changes. (2) The neutralization between Ag NPs and bacterial surfaces changes the cell-membrane permeability, resulting in bacterial death. (3) Ag NPs can bind to cell membrane proteins and intracellular components (lipids, proteins, and DNA), which could also affect transcription, translation, and glucose metabolism. (4) Ag NPs produced by oxidative solubilization can block the transport and release of K^+^ and prevent the synthesis of adenosine triphosphate [81,82,83,84].

Ren et al. added Ag NPs into a CS/konjac glucomannan hydrogel to obtain the NMFH that showed great prospects as a safe wound dressing [85]. This hydrogel matrix acted as a carrier to avoid the burst release of Ag NPs and reduce their cytotoxicity, achieving continuous release to inhibit the inflammatory response. In addition, it was reported that the doping of TiO_2_ with a small amount of Ag could improve not only the antibacterial activity, but also the mechanical properties [86,87]. As an example, Ag-doped TiO_2_ (Ag/TiO_2_) NPs were synthesized by a chemical reduction process and then dispersed in a poly(vinyl alcohol) (PVA) matrix to obtain a hydrogel through a repeated freeze-thawing process. This hydrogel exhibited good antimicrobial activity due to the light-induced ROS and significantly accelerated wound healing [88]. Zhang and Wu et al. utilized Ag NPs as an antibacterial enhancer, introducing them into a matrix based on cationic chitosan and anionic dextran to develop an antifouling hydrogel [89]. The ratio of the two polymers was optimized to achieve a near zero potential for the hydrogel, thus endowing it with an antifouling property. In addition, the introduction of Ag NPs endowed the hydrogel with continuous and broad-spectrum antibacterial activity. Furthermore, Ag oxides have also been proven to have antibacterial activity. Mohammadimehr et al. loaded Ag_2_O/SiO_2_ and calendula officinalis flower extract in a CS/sodium alginate (SA)/PVA hydrogel, which showed great antibacterial activity and potential to promote wound healing [90]. Besides, various types of composite hydrogels employing Ag NPs in synergy with other types of NMs as antimicrobial components have been reported, such as Ag NPs combined with g-C_3_N_4_/TiO_2_ [91], melatonin [92], and GO [86].

**Figure 5:**
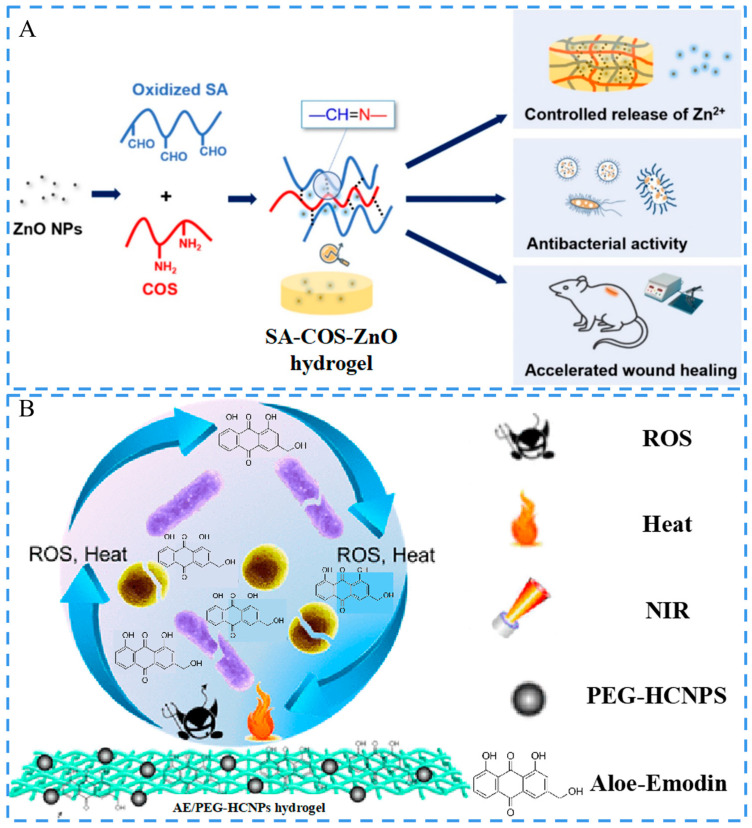
Schematics of the antimicrobial hydrogels. (**A**) Schematic of the construction of SA-COS-ZnO hydrogel with controlled release of Zn^2+^, antibacterial activity, and accelerated wound healing. Reproduced with permission [95]. Copyright 2021, Elsevier. (**B**) Schematic of the function of the AE/PEG-HCNPs hydrogel. The hydrogel exhibited a photothermal effect with ROS scavenging ability. The AE showed antimicrobial activity against G+ and G- bacterial. Reproduced with permission [102]. Copyright 2018, The American Chemical Society.

Except for Ag NMs, other metal NMs also showed good antimicrobial activities against lethal viruses and microorganisms [93]. Numerous studies have demonstrated that ZnO has high antimicrobial activity and that it can be applied to cutaneous wound treatment [94]. Zhao et al. added ZnO NPs to a sodium alginate-chitosan oligosaccharide (SA-COS-ZnO) hydrogel [95] (Figure 5A). The isoelectric point of ZnO NPs is 9–10, and H^+^ from the environment would transfer to the surface of ZnO NPs under physiological pH conditions, resulting in a positively charged surface (ZnOH^2+^) and the dissolution of Zn^2+^. Trace Zn could promote the formation of neovascularization, and Zn^2+^ had antibacterial activity, which led to tissue healing and recovery. Additionally, some other common metal-based materials can also be utilized for the development of antimicrobial materials, such as Au, Cu, and Fe [96]. Zhang et al. prepared a hydrogel with excellent antibacterial ability by loading Au NPs into a heparin/PVA matrix. This hydrogel-based dressing exhibited strong antibacterial activity against Gram-positive (G+) and Gram-negative (G−) bacteria due to the introduction of Au NPs [97]. Zare and Makvandi et al. constructed a hydrogel by incorporating CuO into a sodium carboxymethylated starch network via a solution-casting technique [98]. CuO NPs were proved to have antioxidant activity because of the hydroxyl groups present on their surfaces, thus being used for the relief of oxidative stress at a wound site. The antibacterial activity of the CuO NPs was attributed to ROS generation by the released metal ions, which caused malfunction of the bacteria cell membranes. Moreover, CuO in the hydrogel exhibited excellent antibacterial and antioxidant activities, which effectively promoted wound healing. Ren and Tang et al. co-embedded glucose oxidase (GOx) with Fe_3_O_4_/TiO_2_/Ag_3_PO_4_ nanocomplexes in a poly(acrylic acid) (PAAc)-calcium phosphate hydrogel by in situ biomimetic mineralization. This complex system functioned as a controllable ROS generator and demonstrated extraordinary antimicrobial activity in both in vitro and in vivo studies [99].

Furthermore, some non-metallic NMs also exhibit remarkable antimicrobial properties. Quercetin NPs (Qu NPs) are natural flavonoids with prominent anti-inflammatory effects that can react with polyhydroxy polymers via unreacted neighboring diols [100]. Wang et al. selected PVA as the hydrogel matrix to develop a Qu NPs-containing composite hydrogel, which exhibited excellent antimicrobial and antioxidant activities. The obtained hydrogel film could significantly promote full thickness wound healing [101]. Furthermore, carbon-based materials are also the choice of many researchers for the construction of antibacterial hydrogel dressings. Fan et al. constructed a wound dressing by co-embedding PEG-grafted hollow carbon NPs (PEG-HCNPs) and aloe rhodopsin (AE) in a poly(2-dimethylaminoethyl methacrylate)-based hydrogel [102]. This hydrogel could maintain long-term antimicrobial activity in combination with a photothermal effect (Figure 5B). Pamuła and Reczyńska-Kolman et al. prepared lipid NPs enriched with nisin and introduced them into a hydrogel based on gellan gum and Alg. The hydrogel ensured an extended release of the active ingredients, thus improving the antimicrobial activity [103]. Lin, Qu, and Liu et al. designed an NMFH based on reduced PDA NPs (rPDA NPs) and PEG [73]. The hydrogels were formed through crosslinking among rPDA NPs, 4-arm-PEG-succinimidyl carboxymethyl ester (4-arm-PEG-SCM), and 4-arm-PEG-NH_2_ through an amide reaction. The pre gel solution could solidify quickly within 1 s to form a barrier to achieve antifouling. Moreover, due to the photothermal effect of rPDA NPs, the hydrogel was able to realize effective bacterial eradication under the irradiation of near-infrared light (NIR).

### 3.3. Conductivity

Skin is sensitive to electrical stimulation (ES). The electrical signal plays an important role in maintaining tissue function and the regeneration [104]. Therefore, conductive hydrogels with optimal electrical transmission properties and the ability to interact with tissues are advantageous for soft tissue regeneration. Since the 1990s, researchers have been exploring the application of ES therapy to treat skin wounds [105,106,107]. It was demonstrated that the introduction of an external direct current electric field could accelerate the recovery of skin structure and function and regulate the migration and proliferation of cells by activating ion channels and downstream transduction signals [108,109]. In addition, the ES can also promote neovascularization and modulate the inflammatory response [110]. As a result, conductive hydrogels are in the spotlight in cutaneous wound treatment [111], in which the NMs that have been employed for the development of conductive hydrogels can be grouped into three categories: carbon-based materials, metals, and conductive polymers.

**Figure 6:**
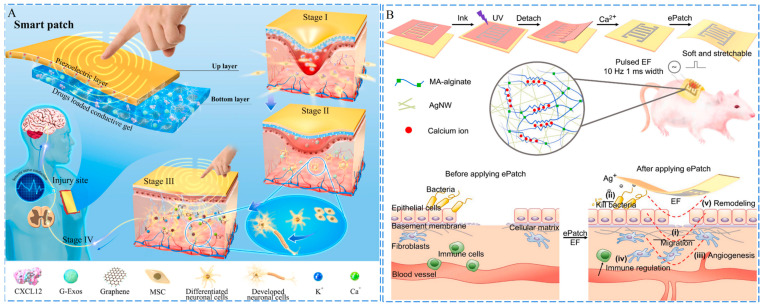
Schematics of the conductive hydrogels. (**A**) Schematics of the hierarchical architecture of the smart patch and its stimulation in endogenous stem cells based neural restoration and sensory function recovery. Adapted with permission [118]. Copyright 2022, Elsevier. (**B**) Schematics of the fabrication of ePatch and five biological activities of the ePatch during the healing process after being applied to wounded SD rats. Adapted with permission [119]. Copyright 2022, Elsevier.

Carbon nanotubes (CNTs) are suitable carbon-based conductive materials because they have a larger specific surface area and higher conductive efficiency than conventional conductive materials [112]. For instance, a composite conductive hydrogel for wound healing was constructed by introducing conductive complexes and carboxylate multiwalled carbon nanotubes (MWCNTs) in the gel matrix. In addition, the hydrogel also had the ability to monitor the movement of the wound, such as the change in charge of conductive material caused by the change of stress in joints or elbows [113]. Guo and his team have developed a series of conductive hydrogels based on carbon-based materials for various wounds treatment, including DA-gelatin/CS/PDA@CNTs composite hydrogel utilized for treatment of infected wound [37], *N*-carboxyethyl CS/benzaldehyde-terminated PF127/CNTs composite hydrogel used for treatment of infected full-thickness skin wound [114], and quaternized CS-graft-cyclodextrin/quaternized CS-graft-adamantane/GO-graft-cyclodextrin composite hydrogel applied for treatment of skin defects [115]. Additionally, nerve damage frequently occurs in conjunction with cutaneous wounds. Electrical signals have been found to effectively stimulate nerve repair by inducing mesenchymal stem cells to differentiate into neural cells [116,117]. Peng et al. constructed a self-powered smart patch via coating of GelMA@rGO hydrogel onto polyvinylidene fluoride (PVDF) film [118]. The PVDF film acted as a flexible piezoelectric generator for electrical stimulation, while the hydrogel served as the reservoir of chemokine and neural directing exosomes as well as the electrode to transfer electric cues. The patch could promote nerve regeneration and sensation restoration at the wound site within 23 days, exhibiting great potential in nerve-damaged wound treatment (Figure 6A).

Conductive hydrogels create conditions for the introduction of exogenous currents. The current parameters of the conductive layer are adjustable in the application. Ag-based NMs are good candidates for the development of conductive hydrogels. Khademhosseini et al. used Ag nanowires (NWs) as the conductive substance and methylarsonic acid (MAA) as the binder to prepare a conductive ink [119]. Figure 6B illustrates that this ink was then cast and shaped in a mold to form a conductive hydrogel patch via photocrosslinking, which achieved rapid wound healing after the addition of Pulse Width Modulation. Chen and Zhou et al. considered Ag NPs as the conductive material and used lignin as the matrix to prepare a composite hydrogel. The lignin could reduce Ag^+^ in situ based on its natural reducing ability. Furthermore, the hydrogel could form a quinone/catechol structure with a dynamic redox environment, and lignin could be modified with hydroxypropyl cellulose and phenylboronic acid by a dynamic borate bond. This hydrogel is able to accelerate epithelial tissue regeneration and neovascularization, reduce inflammatory cell infiltration, promote collagen (COL) deposition, and promote M_2_ macrophage polarization [120]. Moreover, Zhou and Zhang et al. prepared a composite hydrogel through loading Ti_3_C_2_T_x_ MXene@PDA NSs in an oxidized HA/poly(glycerol-ethylenimine) (PGE) hydrogel matrix [121]. The nanocomposites endowed the hydrogel with excellent conductivity, improving the quality of wound healing and skin regeneration by constructing a cellular communication network through increased electrical transmission. In addition, this hydrogel also showed great adhesive properties and antimicrobial activity, which synergized with electrical conductivity to promote the healing of infected wounds and the remodeling of their appendages.

Recently, conductive polymers such as polypyrrole (Ppy) and PANI have also been increasingly used to fabricate hydrogel-based dressings. Yu and Zhou et al. prepared an oxidized chondroitin sulfate modified Ppy NPs/GelMA hydrogel with conductive ability. The hydrogel could upregulate the phosphorylation expression of the PI3K/AKT and MEK/ERK pathways by increasing the intracellular Ca^2+^ concentration. Besides, this hydrogel could also promote the migration and axon growth of PC12 neuronal cells at the cellular level and promote the regeneration of the neurovasculature and COL deposition in diabetic wound [122]. Zhang, Tao and Zhu et al. developed an E-skin patch with PF127 serving as the hydrogel matrix, and Ppy serving as the conducting medium [123]. The Ppy provided excellent photothermal and charge transport capabilities for the hydrogel due to its inherent non-radiative relaxation and conjugated structure [124,125]. The hydrogel could promote angiogenesis, COL deposition, and re-epithelialization in combination with PTT and real-time ES effectively. Moreover, Zhang et al. developed a PAAc-sulfonated HA-PANI conductive hydrogel with ES, which exhibited a powerful killing effect on G+ bacteria through specific binding to bacterial lipid calcium phosphate [126]. Unlike the macromolecular form of aniline polymorph, Guo et al. selected the oligomeric form of aniline (aniline tetramer) as the conductive matrix and synergized with oxidized HA and *N*-carboxyethyl CS to construct an injectable conductive hydrogel [127]. The pre gel solution could gelate rapidly under physiological conditions. Additionally, the hydrogel was also modified with amoxicillin to improve its antibacterial activity.

### 3.4. Regulation of ROS Level

ROS are a class of highly reactive chemicals derived from molecular oxygen, mainly including superoxide radicals, hydroxyl radicals, hydrogen peroxide, and singlet oxygen [128]. Moderate levels of ROS can kill bacteria by destroying proteins, lipids, and other components [129,130]. However, the overexpression of ROS will lead to damage to protein and DNA, which may cause cell death and complications like inflammation and fibrosis. Besides, oxidative stress will inhibit cell migration and proliferation and deficiency of GFs [131,132,133,134,135]. Therefore, one effective way to regulate wound healing is to control the level of the ROS [136].

**Figure 7:**
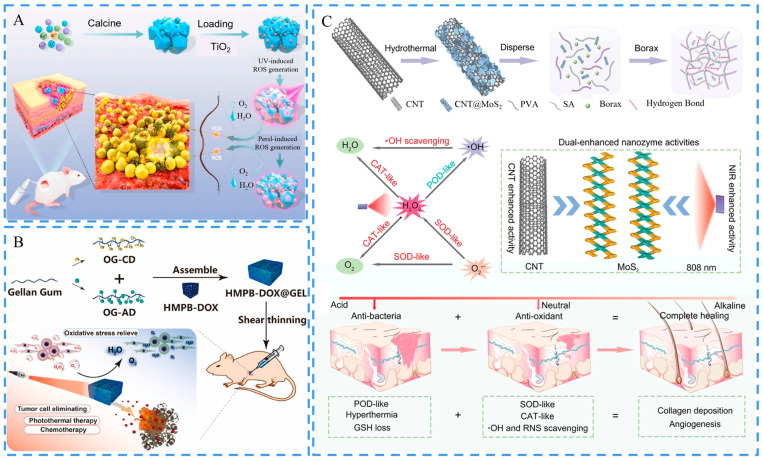
Schematics of the ROS-regulating hydrogels. (**A**) Preparation of the antibacterial photocatalyst LGG/TiO_2_-PF127/PLys/oxidized HA-based hydrogel and its application in infected wound healing. Reproduced with permission [139]. Copyright 2021, Elsevier. (**B**) Schematic of the supramolecular hydrogel containing HMPBs loading doxorubicin (HMPB-DOX@GEL) and its application for tumor postoperative treatment. Adapted with permission [142]. Copyright 2022, Elsevier. (**C**) Schematics of the preparation procedure for the CNT@MoS_2_ NSs incorporating PVA/SA hydrogel and the summary of CNT and NIR dual-enhanced triple nanozyme activities. Reproduced with permission [143]. Copyright 2021, Wiley-VCH.

Metals and their oxides are the choice of many researchers for the construction of hydrogel-based dressings with the ability of ROS level regulation. Xie et al. creatively developed an Ångstrom-scale Ag particles-loaded carbomer gel for the treatment of diabetic wounds and large burns. The Ångstrom-scale Ag particles were significantly smaller in scale than commercial Ag NPs and exhibited enhanced ROS generation capacity, effectively inhibiting bacterial colonization and inflammatory response level [137]. TiO_2_ is another kind of metal oxide that has both acoustic and photo-sensitivities. You and Yu et al. constructed a gel of amorphous TiO_x_ nanofibers dotted with Ti_2_C(OH)_2_ NSs [138]. This hydrogel could trap the bacteria by forming a Ti-O-P bond between the phosphate of the bacterial cell wall and Ti-OH, and it could generate sufficient ROS to kill bacteria by combining with exogenous ultrasound radiation. Meanwhile, Ti_2_C(OH)_2_ in the gel converted H_2_O_2_ to O_2_ and accelerated wound healing. TiO_2_ has also been used for antimicrobial photocatalytic therapy. Zhang et al. prepared PF127, PLys, and oxidized HA-based hydrogel with antimicrobial photocatalytic activity by combining TiO_2_ NPs and LiLuGeO4:Bi^3+^ (LGG) with a UV persistent light-emitting properties [139]. After being stimulated by UV light, LGG emitted light that TiO_2_ absorbed to produce persistent ROS, exhibiting long-lasting antimicrobial activity (Figure 7A).

As mentioned above, excessive ROS can cause damage to cell membranes and proteins, impeding tissue regeneration during wound healing processes. Hydrogels with antioxidant capacity are able to control the inflammatory response and accelerate wound healing. Interestingly, Ag NPs can also play an important role in antioxidants. Wang et al. loaded Ag NPs into a DA-gelatin/guar gum-based hydrogel. The obtained hydrogel had excellent antioxidant ability and could scavenge ROS in the wound microenvironment to improve the efficiency of the wound treatment [140]. Nanozymes are NMs that mimic natural enzymes in their reaction mechanism [141]. Ding and Mao et al. prepared a supramolecular hydrogel loaded with hollow mesoporous Prussian blue NPs (HMBPs) [142]. This hydrogel was formed with cyclodextrin (host) and adamantane (guest) modified gellan gums through host-guest interaction. HMBPs were utilized for PTT and to scavenge overexpressed ROS, while the hybrid hydrogel exhibited POD-like activity and SOD-like activity under light irradiation, efficiently relieving oxidative stress and promoting wound healing (Figure 7B). Zhu and Fan et al. developed a gel based on CNTs loaded with MoS_2_ NSs. The nanocomplex showed three enzyme-like activities [143]. This hydrogel exhibited peroxidase (POD) activity in an acidic environment and showed superoxide lyase activity and catalase (CAT) activity in a neutral condition. These bioactivities efficiently convert superoxide radicals to H_2_O_2_ and O_2_ and decompose H_2_O_2_ to O_2_. In addition, the photothermal activity of CNT enhanced the catalytic effect of MoS_2,_ and the resulting hydrogel exhibited good free radical scavenging ability (Figure 7C). CeO_2_ exhibits excellent ROS redox quenching activity due to the richness of oxygen defects and Ce^3+^ species [144]. Krebs et al. reported a zwitterionic hydrogel by combining CeO_2_ NPs with microRNA, which has anti-inflammatory activity. In this system, CeO_2_ NPs acted as an antioxidant to scavenge overexpressed ROS and reduced the level of the inflammatory response by combining with microRNA [145]. Many non-metallic NMs also exhibit great antioxidant potential. Wu and Wang et al. developed a hydrogel using PDA NPs and two natural antioxidant drugs (puerarin and ferulic acid) based on a “the more natural the better” strategy [146]. Under oxidative stress conditions, the hydrogel enhanced superoxide dismutase (SOD) and glutathione peroxidase activities, efficiently scavenged ROS, and reduced malondialdehyde levels. Consequently, the hydrogel circumvented the damage caused by overexpressed ROS. Lu et al. developed a scaffold by incorporating PDA-rGO into a CS/SF-based matrix (pGO-CS/SF) for synergistic antioxidant therapy [147]. The developed bio-scaffold reduced cellular oxidation by removing excess ROS. Meanwhile, this scaffold responded to electrical signals and promoted physiological electrical signals for cell growth to improve tissue regeneration in the wound.

### 3.5. Stimulus Responsiveness

#### 3.5.1. Photo-Responsiveness

In a spatio-temporal manner, light can regulate some behavior of photo-responsive hydrogels. An adjustable light source with tunable wavelength, power, and duration can be considered a switch to trigger heat production, degradation, or specific biological functions of the hydrogels [148,149].

**Figure 8:**
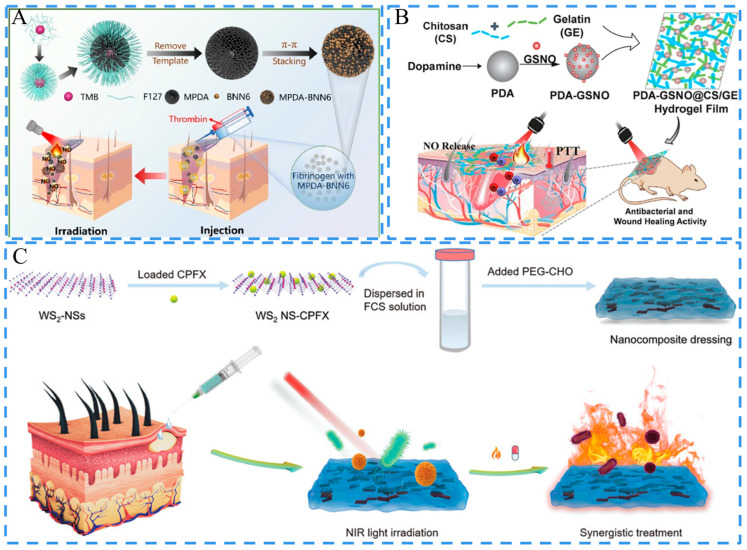
Schematics of the photo-responsive hydrogels. (**A**) Schematic of the fabrication and application of the hydrogel MPDA-BNN6@fibrin in anti-infective therapy and wound healing. Reproduced with permission [160]. Copyright 2022, Elsevier. (**B**) Schematic of synthetic procedure and NIR-triggered PTT and NO therapy of the hydrogel PDA-GSNO@CS/gelatin for enhanced wound healing. Adapted with permission [161]. Copyright 2022, Elsevier. (**C**) Schematics of the preparation procedure for WS_2_-CPFX/FCS/PEG-CHO hydrogel and the synergistic therapy of an infected wound. Adapted with permission [172]. Copyright 2020, Elsevier.

Photo-responsive hydrogels can be fabricated by introducing NMs with light responsiveness into the gel network [150]. Photothermal therapy (PTT) is a new and emerging treatment method. After the irritation, the absorbed optical energy converts to thermal energy, resulting in the ablation of bacteria via quickly raising the temperature [151]. Additionally, photodynamic therapy (PDT) is based on the photooxidation of the photosensitive dye in the target tissue for the treatment of a wide variety of diseases [152]. It produces few adverse effects and requires a short treatment period [153]. In recent studies, PDA NPs, quantum dots (QDs), porphyrin-based NPs, and some metal-based NPs have been used to construct photo-responsive hydrogels [154,155,156]. For instance, PDA NPs have been widely utilized in the field of light-related therapies due to the strong light absorption and photothermal conversion efficiency [157,158]. In addition, PDA NPs have also been applied in the development of adhesive hydrogels due to their catechol structure [159]. Yang, Tu, and Dong, et al. encapsulate *N*,*N*′-disec-butyl-*N*,*N*′-dinitroso-*p*-phenylenediamine(BNN6)-loaded mesoporous polydopamine (MPDA) in a fibrin gel (MPDA-BNN6 NPs@fibrin) for the treatment of an infected wound [160]. Under the action of thrombin, soluble fibrinogen was converted into the insoluble fibrin gel after fibrinopeptides were resected from fibrinogen. The hydrogel acted as a light-operated nitric oxide (NO) generator and achieved photothermal/gas dual-modal precise antibacterial therapy under the irradiation of 808 nm light to accelerate bacterial-infected wound closure (Figure 8A). Yang, Ding, and Chen et al. also adapted the strategy to construct a CS/gelatin-based hydrogel with photothermal properties and gas releasing ability [161]. PDA NPs were selected as the photothermal agent, and *S*-nitrosoglutathione (GSNO) was utilized to endow the hydrogel with NO releasing ability. The hydrogel exhibited a local thermal effect and released NO in response to NIR stimulation, resulting in a synergetic strategy for infected wound treatment (Figure 8B). Lu et al. combined PDA NPs with a SF/CS hybrid matrix to construct a bionic gel. In the system, the active catechol groups on the PDA NPs provided multiple bioactive sites for protein and peptide adsorption. The composite gel exerted a potent bactericidal effect under NIR irradiation, thereby modulating the inflammatory response and inhibiting bacterial invasion for wound healing [162]. Li, Huang, and Wu et al. added PDA NPs, GOx, and CAT into a PDA/PAM hydrogel for refractory diabetic wounds. The PDA NPs exhibited an efficient antimicrobial effect under NIR irradiation. The hydrogel could also effectively control blood glucose levels at the wound site due to the introduction of GOx and CAT. Furthermore, the hydrogel could produce sufficient oxygen to support cell proliferation [163].

Carbon quantum dots (CQDs) are another photothermal conversion agent with photoluminescent and free radical scavenging properties [164]. Wu et al. constructed a photo-responsive hydrogel by introducing CQDs and ZnO into a folic acid-conjugated PDA composite system [165]. CQDs could not only generate ROS rapidly but also exert inherent photoresponsivity to achieve 99.9% killing of G+ and G- bacterial under 660 nm and 808 nm light illumination. The Zn^2+^ released from the hydrogel also provided a long-lasting antibacterial property and regulated cell behavior by promoting the proliferation of fibroblasts. Das et al. constructed a hydrogel via loading DNA-ligated CNDs into a poly(vinylpyrrolidone) matrix, and the CNDs generated ROS when visible light irradiation for efficient sterilization. In addition, the hydrogel could be used to track drug delivery because the fluorescence of CND would burst in the presence of a heme [166].

Nanozymes have also been applied in photosensitization therapy. Two dimensional MOF nanozymes with ultrathin nano-thickness usually have higher catalytic activity than conventional nanozymes [167]. Wang et al. developed a PEG-based hydrogel loaded with 2D zirconium-ferrocene MOF NPs in combination with PTT for skin wound treatment. The hydrogel exhibited potential synergistic antibacterial effects for the treatment of a bacterially infected skin wound [168]. MoS_2_ NM is a 2D material with good biocompatibility and POD-like properties that shows the potential for non-invasive antimicrobial therapy [34]. Additionally, the nano forms of many transition metal elements also have good photo-responsive properties. Cu NPs have a localized surface plasmon resonance effect and can effectively convert NIR irradiant power into local thermal energy for PTT purpose [169]. Cai et al. developed a hydrogel via loading Cu NPs in a matrix based on a copolymer of GelMA and *N*,*N*-bis(acryloyl)-cystamin. The Cu NPs embedded in the hydrogel produced ROS and converted the excitation energy of NIR into local heat, demonstrating a powerful killing ability against both G+ and G- bacteria [170]. Au and Ag also present the surface plasmon resonance effect. Zhou et al. prepared a hydrogel with photothermal effects by loading Au and Ag hybrid NSs into SH. After laser irradiation, AgAuNSs eradicated *E. coli* and *S. aureus* [171]. Yu et al. co-added WS_2_ NSs with the antibiotic ciprofloxacin (CPFX) into a matrix based on dodecyl-modified chitosan (FCS) and dialdehyde-functionalized PEG (PEG-CHO) to construct a hydrogel with a photothermal effect and spatio-temporal modulation of drug release [172]. WS_2_ generated a large amount of heat under NIR irradiation, which simultaneously triggered the release of antibiotics. The hydrogel and NIR synergistically promoted wound healing and circumvented the shortcomings of the two separate treatment modes (Figure 8C).

#### 3.5.2. pH Responsiveness

Every biochemical reaction needs to be carried out at an optimal pH. The pH value at the wound site changes dynamically during the healing process, which means the state of wound healing can be monitored and regulated by manipulating the local pH [173]. The optimization of wound healing speed by artificially introducing NMs capable of regulating pH has been studied [174].

**Figure 9:**
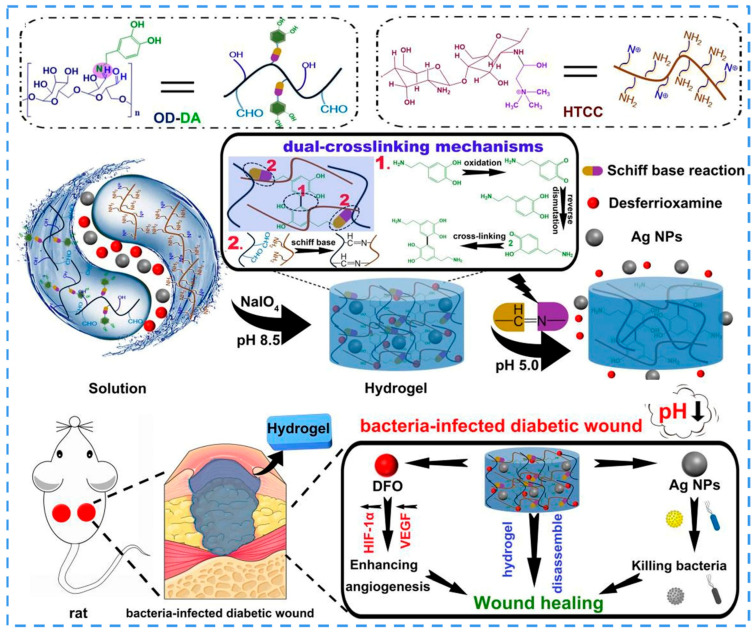
Schematics of the formation and mechanisms of hydrogel Ag NPs@HTCC/ODEX-DA (ODEX-DA = OD-DA), and the antibacterial and wound healing mechanisms including enhancing angiogenesis and killing bacteria. Adapted with permission [178]. Copyright 2021, Elsevier.

Most bacteria produce acetic and lactic acids during multiplication and metabolism in infected wounds, resulting in local acidification (pH 4.5–6.5) [175]. Srivastava et al. loaded a polyelectrolyte-vitamin C nanoparticulate system into a PVA-Alg hydrogel to create an acidic environment around the wound to promote faster closure [176]. Dai and Li et al. loaded Ag NPs in a lignocellulose/SA/PVA network to construct a hydrogel. The acidic environment led to the disruption between Ag and boric acid, generating a longer pH-dependent release behavior of the hydrogel [177]. Wang et al., constructed a dual-cross-linked hydrogel from chitosan, quaternary ammonium salt, and ODEX-DA through the formation of catechol-catechol adducts and the Schiff base reaction [178]. They also opted for Ag NPs as the antimicrobial agent against infectious bacteria in the diabetic wound microenvironment. The acidic environment disrupted the network structure, leading to the responsive release of Ag NPs (Figure 9). Copello et al. developed a keratin hydrogel loaded with ZnO NPs for protection against bacterial infections. The hydrogel shrank under acidic conditions and swelled in response to contact with alkaline media in chronic wounds, thereby releasing NPs that formed an antimicrobial barrier and protected the wound from bacterial infection [179].

Changes in pH can regulate the release of the drug by altering the formation and breaking of chemical bonds between the hydrogel network and the drug [180]. Moreover, the gelation and degradation processes of hydrogels can also be controlled by adjusting the pH to manipulate loaded drug release [181]. Zeng et al. constructed an ultrasensitive pH-responsive hydrogel, which formed through the Schiff base reaction between an aldehyde-containing copolymer from 2-methacryloyloxyethyl phosphorylcholine (MPC), 4-formylbenzoate ethyl methacrylate (FBEMA), and amine-modified SiO_2_ NPs. In the neutral environment, the pre gel solution could gelatinize rapidly within 10 s. More intelligently, the hydrogel underwent a rapid gel-sol transition to trigger drug release in an acidic environment created by the acute inflammatory response [182]. Liu, Jin, and Xuan et al. constructed a CS hydrogel encapsulating formyl-met-leu-phe (fMLP) and FasL-conjugated silica NPs (SiO_2_-fasL). This hydrogel degraded when it was applied to the wound site because of the acidic microenvironment. The fMLP recruited neutrophils to the wound to initiate an inflammatory reaction, which created an acidic environment. Then the pH-responsive hydrogel matrix began to degrade, and the exposed SiO_2_-fasL triggered FasL-Fas signaling to induce neutrophil apoptosis, promoting the conversion of the anti-inflammatory phenotype of macrophages to drive regeneration. This creative strategy for transient enhancement of the inflammatory response provided a novel solution for the treatment of diabetic skin wound [183].

#### 3.5.3. Magnetic Responsiveness

**Figure 10:**
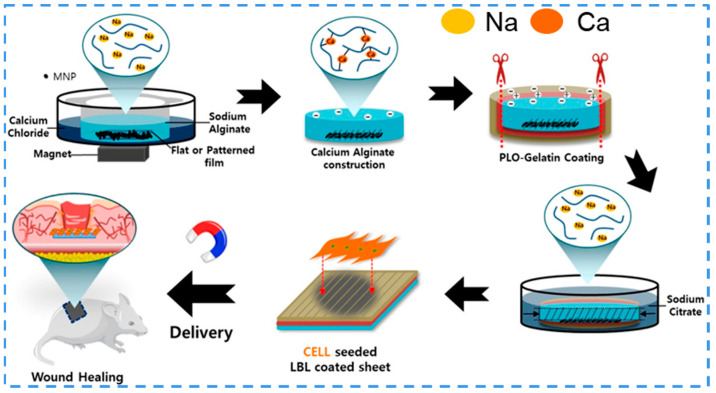
Schematic of the synthesis procedure of MNPs-embedded hydrogel sheet with groove pattern for wound healing. Reproduced with permission [191]. Copyright 2019, The American Chemical Society.

Magnetic responsive hydrogels are highly sensitive to the magnetic field. The integration of magnetic NPs (MNPs) and functional polymer networks can be achieved through electrostatic interaction or hydrogen bonding, such as to Fe_3_O_4_ and MnFe_2_O_4_ NPs, and can be introduced into biopolymer networks [184]. The introduction of magnetic materials into the gel network allows the hydrogel to have remotely tunable properties. Furthermore, the magnetic field is an intermediate medium to regulate the delivery and release of the loaded drug without any additional contact, showing great potential for further development of multifunctional hydrogels in the biomedical engineering field [185,186]. Fe_3_O_4_ NPs are magnetic NPs with superparamagnetic property [187]. Tan and Chen et al. constructed a polysaccharide-based magnetic hydrogel by encapsulating Fe_3_O_4_ NPs in a carboxymethyl cellulose and CS network. The magnetic microspheres enabled the hydrogel to have a fast and strong response to the external magnetic field, offering the possibility of delivering the loaded drug [188]. Forouzandehdel and Rami et al. prepared an itaconic acid/starch/fucoidan-based hydrogel loaded with a magnetic field responsive Fe_3_O_4_@graphene sheets nanocomplex and guaifenesin as a wound treatment drug. The hydrogel could achieve on-demand drug delivery under the action of an external magnetic field [189]. This model was also adopted by Sadeghi et al. They used itaconic acid/starch as a hydrogel matrix and selected Fe_3_O_4_ NMs as the magnetic responsive agent to achieve controlled release of the drug under the action of an external magnetic field [190]. This model provides a universal and pervasive platform for responsive controlled release that can be applied not only in the field of wound healing, but also in the treatment of other diseases, and it is worth further study. In addition, Wang et al. fabricated an Alg/poly-*L*-ornithine/gelatin hydrogel sheet with a groove pattern for use as a cell delivery platform [191]. Fe_3_O_4_ NPs were incorporated into the patterned hydrogel sheet for the magnetic field-induced transfer of cell-seeded hydrogel sheets. In the meantime, cell differentiation from an endothelial progenitor cell (EPC) to an endothelial cell promoted rapid cell migration and vascularization, thus accelerating wound healing (Figure 10). However, the application of NMs-based magnetic hydrogels in wound healing is not yet widespread, probably because wound management is an emergency treatment and the need for drugs or active ingredients is urgent rather than a postponable treatment.

A summary table is provided to introduce the other NMFHs that are not mentioned in the text above (Table 1). Several widely studied types including hemostatic, antimicrobial, conductive, and photo-responsive hydrogels are summarized from NMs species, hydrogel matrix, and the mechanisms of specific biological functions.

## 4. Conclusions and Perspectives

Hydrogels for the treatment of cutaneous wounds have shown tremendous growth in recent decades, and the nanomaterials functionalized hydrogels (NMFHs) perform better in this area. The excellent physical, chemical, and biological properties of nanomaterials (NMs) provide a wide range of opportunities for the targeted functionalization of hydrogels. As one of the most conventional and convenient modification methods, loading NMs can confer diverse functions on the hydrogels, such as hemostasis, antimicrobial activity, conductivity, and the ability to regulate ROS levels. In addition, with the rise of smart hydrogels in recent years, NMs are playing an increasingly important role in the development of stimulus-responsive (pH responsive, photo-responsive, and magnetic responsive) hydrogels. The development of NMs-based stimulus-responsive hydrogels is valuable because such intelligent material can modulate behaviors of the hydrogels by exogenous modifiable means, allowing the release of the internal active ingredients on demand [218,219,220,221]. Precisely, programmed administration is more beneficial for active ingredient release [222].

However, there are some challenges to the development of NMFHs in the wound dressing field, where the creation of multifunctional hydrogels is meaningful when applying them to complex wound scenarios. Hence, it is necessary to select modifiers that can perform multiple biological functions, mobilize the body’s own repair potential, and activate the production of endogenous active substances to achieve better healing results. In these scenarios, the hydrogels can be artificially modified by selecting cell-derived proteins, such as growth factors (GFs), chemokines, or nucleic acid sequences with specific biological functions to be grafted onto the NMs. Secondly, the biocompatibility of the hydrogels should be further improved, and systematic evaluations need to be performed to promote their clinical applications. Thirdly, the mechanical properties of some hydrogels should be improved to ensure the healing efficiency of the materials during the whole healing process. The process of wound healing is complex, including variations in mechanical properties and cell behaviors. Moreover, tissue structure and mechanical forces are important signals for cell decision making, and the forces are constantly transferred between the extracellular machinery matrix and intercellular adhesion sites. The key is the mechanical changes caused by the cell and the ECM, as well as the forces generated in the cell and exerted by the outside world [223,224]. Therefore, NMFHs with controllable mechanical strength are desirable. Besides, stem cell therapy is an emerging therapy for cutaneous wound treatment, and its application to wounds based on NMFHs is a bright prospect. Stimulus-responsive NMFHs may be able to regulate the fate of loaded stem cells under physicochemical stimulations, thus avoiding the inevitable side effects caused by the introduction of exogenous proteins or chemical inducers [225,226].

In conclusion, NMFHs have significant research and application values in skin wound dressings. To address the expanding and complex problem of wound repair and lessen the suffering of patients, cutaneous wound hydrogel dressings will be developed in the future with improved biocompatibility, better repair effects, and a higher degree of bionic mimicry. It is hoped that this review will inspire researchers to generate more creative ideas and concepts in the field of NMFHs for cutaneous wound healing.

## Figures and Tables

**Table 1:** Summary of other NMFHs with different functions.

Function	Nanomaterial	Hydrogel Matrix	Mechanism	Ref
Hemostatic Ability	polydopamine decorated Ag NPs (PDA@Ag NPs)	Oxidized Alg and catechol-modified gelatin	Good adhesion reduced blood loss and gelatin promoted platelet aggregation.	[192]
	GO	dopamine grafted gelatin (GelDA)	The catechol group of dopamine (DA) attached to wound and stopped bleeding.	[193]
	transferrin conjugated CuO_2_ NPs (CP@Tf NPs)	copolymer of *N*-isopropylacrylamide (NIPAM), acrylamide (Aam), *N*-[3-(dimethylamino)propyl]-methacrylamide (DMPA), and methylene-*N*,*N*-bis(acrylamide)	Abundant amino acid groups of the hydrogel attracted negatively charged red blood cells to gather and form blood clotting, and Cu^2+^ promoted coagulation.	[194]
	Au NPs	CS	Au NPs stimulated the intrinsic coagulation pathway.	[195]
	multiwalled carbon nanotubes (MWCNTs)	copolymer of glycidyl methacrylate functionalized quaternized-CS (QCSG) and PF127	MWCNTs trigger platelets activation and Ca^2+^ from extracellular activated the release of platelet membrane microparticles.	[196]
	nano whitlockite (nWH)	CS	Various coagulation factors involved in the coagulation cascade were activated by the Ca^2+^, Mg^2+^, and PO_4_^3−^ released from nWH and amine groups of CS.	[197]
Antimicrobial activities	Ag NPs	galacto-xyloglucan and PAM	The released Ag^+^ affected the replication and/or inactivation of the microbial flora.	[198]
	PDA@Ag NPs	PANI and PVA	Ag NPs released Ag^+^ and bind to bacteria to destroy them.	[199]
	Zn doped nWH (Zn-nWH)	copolymer of methacrylate anhydride quaternized CS (QCSMA) and methacrylate anhydride DA (DAMA)	The Zn^2+^ released from Zn-nWH synerging with QCSMA achieved a high antibacterial effect.	[200]
	Cu NPs	CS/ PF127	Depolarization of the cell membrane through interaction between the cell membrane and Cu NPs weakened the cell outer membrane, and Cu^2+^ penetrated the cell and mediated the ROS to block the bacterial cell metabolism.	[201]
	GO/CuO nanocomposite	CS and PVA	NPs accumulate around bacteria, causing bacterial oxidative stress, DNA damage, and lactate dehydrogenase (LDH) release.	[202]
	Ag NPs	copolymer of *L*-DA, PEG, and gelatin (GPLD)	Star-shaped topology cationic GPLD with and/or certain functional group on the side chain showed antimicrobial activity.	[203]
	TiO_2_ NPs or Ag NPs	xylan and CS	Incorporation of TiO_2_ NPs or Ag NPs in the gel matrix provided synergistic effects in killing bacterial.	[204]
	CeO NPs	DA-modified GelMA	CeO NPs cleaned extracellular ROS and prevented intracellular ROS production.	[205]
	Ag NPs	silk fibroin (SF)	Ag NPs destroyed the bacterial structure and inhibited the inflammatory response.	[21]
	Ag NPs	porcine dermal decellularized extracellular matrix	Ag NPs destroyed the structure of bacteria.	[206]
	reduced graphene oxide (rGO)	DA modified HA (DA-HA)	High temperature (above 50℃) could kill bacteria through destroying some enzymes and proteins.	[8]
Conductivity	CNTs	GelMA	The conductive hydrogel could promote the NE-C4 stem cells proliferation and differentiation.	[207]
	CNTs	DA-gelatin/CS/PDA	The conductive hydrogel could adjust electrical signals and promote wound healing via improving blood flow, enhancing migration, and reducing edema.	[37]
	CNTs	*N*-carboxyethyl CS/benzaldehyde-terminated PF127	The CNT-based conductive hydrogel showed photothermal ability to shorten the healing process of infected wound.	[114]
	GO-graft-cyclodextrin	quaternized CS-graft-cyclodextrin/quaternized CS-graft-adamantane	The hydrogel could regulate cell adhesion, proliferation, and migration with/without ES.	[115]
	TA-chelated Ag NPs	PAAc	The conductive hydrogels facilitated the earliest stage of myotube formation.	[208]
	Ppy NPs	GelMA/CS-catechol	The conductive hydrogel could regulate cellular behavior and benefit better integration and growth with tissues.	[209]
	Ti_3_C_2_T_x_ MXene@CeO_2_ nanocomposites	Polyethyleneimine grafted-PF127/oxidized SA	The conductive hydrogel is beneficial to the proliferation and migration of fibroblasts under the ES.	[210]
Photo-responsiveness	ZnO QDs@GO	CS	ROS, and the Zn^2+^ released from ZnO QDs under acid environment killed the bacteria.	[211]
	Cu, N-doped carbon dots (Cu, N-CDs)@GO NSs	CS	The hydrogel could absorb 808 nm light and convert the energy into thermal energy due to the photothermal effects of Cu, N-CDs, and GO NSs.	[212]
	UiO-66-NH_2_ MOF NPs	CS	The MOF could produce active oxygen (·OH) by pre-UV-irradiation or in the presence of a trace amount of H_2_O_2_ to kill the bacteria.	[213]
	berberine chloride NPs	*N*-(9-fluorenylmethoxycarbonyl)-L-phenylalanine	The hydrogel exhibited AIE behavior and ^1^O_2_ generation under white light, and kill bacteria via a PDT mechanism, and in turn penetrate and eradicate biofilms.	[214]
	black phosphorus QDs (BPQDs)	PVA/Alg	BPQDs were photo responsive, ROS-generating, and antibacterial, which could promote the MRSA-infected wound healing.	[215]
	catechol-modified CS-derived carbonized polymer dots (CPDs)	PVA	The PVA@CPDs hydrogel could reach the desired temperature quickly under irradiation, thus efficiently killing the bacteria and preventing overheating of normal tissues.	[216]
	PANI NPs	PAM	The hydrogel could convert light energy into heat upon NIR irradiation and be used as a photothermal antibacterial material.	[45]
	Ppy NTs	quaternized CS-graft-β-cyclodextrin, adenine	The PTT enhanced wound healing by promoting collagen deposition.	[217]

## Data Availability

Not applicable.
